# Retroviral hijacking of host transport pathways for genome nuclear export

**DOI:** 10.1128/mbio.00070-23

**Published:** 2023-11-01

**Authors:** Ryan T. Behrens, Nathan M. Sherer

**Affiliations:** 1 Department of Pathology and Laboratory Medicine, University of Wisconsin, Madison, Wisconsin, USA; 2 McArdle Laboratory for Cancer Research and Carbone Cancer Center, University of Wisconsin, Madison, Wisconsin, USA; 3 Institute for Molecular Virology, University of Wisconsin, Madison, Wisconsin, USA; Ohio State University, Columbus, Ohio, USA

**Keywords:** virus, nuclear export, nuclear pore complex, nuclear envelope, subcellular trafficking, nuclear membrane budding, Exportin-1, CRM1, NXF1, human immunodeficiency virus, Rev, Rex

## Abstract

Recent advances in the study of virus-cell interactions have improved our understanding of how viruses that replicate their genomes in the nucleus (e.g., retroviruses, hepadnaviruses, herpesviruses, and a subset of RNA viruses) hijack cellular pathways to export these genomes to the cytoplasm where they access virion egress pathways. These findings shed light on novel aspects of viral life cycles relevant to the development of new antiviral strategies and can yield new tractable, virus-based tools for exposing additional secrets of the cell. The goal of this review is to summarize defined and emerging modes of virus-host interactions that drive the transit of viral genomes out of the nucleus across the nuclear envelope barrier, with an emphasis on retroviruses that are most extensively studied. In this context, we prioritize discussion of recent progress in understanding the trafficking and function of the human immunodeficiency virus type 1 Rev protein, exemplifying a relatively refined example of stepwise, cooperativity-driven viral subversion of multi-subunit host transport receptor complexes.

## INTRODUCTION

Nuclear genome replication is intrinsic to the multiplication and spread of reverse-transcribing viruses (e.g., retroviruses and hepadnaviruses), most DNA viruses, and a subset of negative-strand RNA viruses. Each of these viral families is adapted to replicate genomes in the nucleus prior to trafficking their nascent nucleic acid genomes efficiently from the nucleus to the cytoplasm over the course of infection. With the notable exception of orthomyxoviruses and bornaviruses, most RNA viruses avoid the nucleus, replicating genomes in the cytoplasm and often targeting nucleocytoplasmic transport for inhibition as a means to slow or abrogate host cellular antiviral responses [reviewed in references (1, 2)].

The major physical barrier to viral genome nuclear export is the nuclear envelope (NE), consisting of two parallel phospholipid bilayers that run continuously with the endoplasmic reticulum, held together by cytoskeletal elements, and dotted with (typically) hundreds of nuclear pore complexes (NPCs) that provide for the select passage of substrates either entering or being exported from the nucleus [reviewed in references (3, 4)]. At a fundamental level, the NE allows for the physical separation and segregation of major nuclear, DNA-driven events (e.g.*,* DNA replication and transcription) from cytoplasmic, RNA-driven events (e.g., protein synthesis). It follows that nuclear replication protects viruses with DNA or complex double-stranded RNA genomes from cytoplasmic antiviral nucleic acid sensors (e.g., RIG-I, MDA5, or cGAS) that might otherwise activate innate immune defenses [reviewed in references (5, 6)]. Consequently, viruses that exploit the safe harbor of the nucleus for genome replication ultimately face the challenge of traversing the NE to deliver new genomes to the cytoplasm where they gain access to virion egress pathways.

Retroviruses, including the human immunodeficiency virus type 1 (HIV-1), are the best-studied viruses in the context of RNA nuclear export, based in part on their need to generate both spliced and unspliced RNA molecules from a single, full-length, viral RNA species (7). Because retroviral RNA genomes are full-length and thus retain introns, their genomes are necessarily adapted to exploit cellular pathways that allow them to overcome otherwise strong cell-intrinsic blocks to the nuclear export and translation of unspliced, partially spliced, or incorrectly spliced mRNAs; signaled by a lack of exon junction complexes and/or the presence of intron-associated inhibitory elements (8, 9). In this minireview, we summarize the major ways by which retroviral RNA genomes hijack the host cell, contrasting these mechanisms with those utilized by other nuclear viral pathogens. In particular, we emphasize recent progress in understanding the remarkable activities of the HIV-1 Rev protein; an excellent example of how stepwise, cooperative interactions among viral and host determinants are exploited to build and activate functional nuclear export complexes at precisely the right place and time.

## BREACHING THE BARRIER: VIRAL GENOME TRANSIT OUT OF THE NUCLEUS

**Fig 1:**
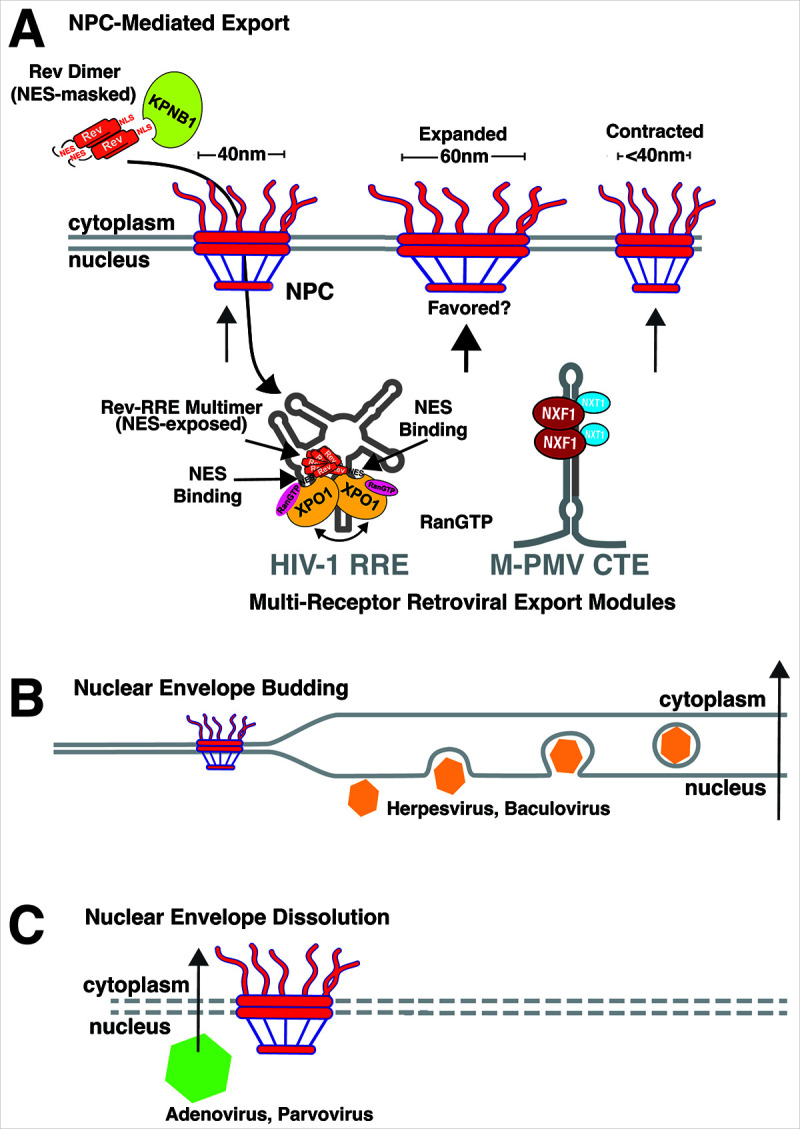
Modes of viral nuclear export. (**A**) NPC-mediated export. Functional Rev/RRE- or CTE-driven export modules form multi-receptor transport complexes. We propose a model for stepwise cooperative interactions wherein Rev dimerizes in the cytosol to mask its NES, allowing for interactions with KPNB1, followed by exposure of the NES in conjunction with RRE-binding and high-order Rev multimerization. Multiple XPO1 proteins are recruited by Rev to the RRE, and the M-PMV CTE recruits multiple NXF1/NXT heterodimers, suggesting that multiple transport receptors are required to overcome size-dependent limitations on vRNP nuclear export. NPC diameter has been shown to be dynamic so that “expanded” channels might be needed to allow for the export of large vRNP transport complexes. (**B**) Nuclear envelope budding. Alphaherpesviruses and baculoviruses encapsidate their double-stranded DNA genomes in the nucleus, with their capsids predicted to be too large to cross using the nuclear pore. Instead, these viruses transit the NE by exploiting membrane dynamics, budding into the inner lipid bilayer of the NE, and then subsequently fusing the transport vesicle with the outer bilayer of the NE to release capsids into the cytosol. (**C**) Nuclear envelope dissolution. Some DNA viruses (e.g., adenoviruses and parvoviruses) encapsidate their genomes in the nucleus and are thought to trigger cytopathic effects that rupture the NE, allowing for nuclear escape. It is also possible that, for viruses that infect dividing cells such as HBV, nuclear genomes may be translocated to the cytoplasm in conjunction with NE dissolution during the process of cell division. Note that protein, RNA, and membrane sizes are relative and not drawn to scale.

To date, there are at least three independent routes that viruses, in general, are known to exploit to circumvent the NE for genome export (outlined in Fig. 1): (i) regulated transit through the NPC [see also references (8, 10–12)], (ii) crossing the NE independently of NPCs using a pathway known as NE budding (13, 14), or (iii) rupturing the NE through induction of virus-triggered cytopathic effects (15, 16). Below, we summarize examples of each pathway and, based on recent data from live cell imaging (17, 18), speculate that a fourth pathway may exist wherein, should infected cells be actively dividing, a viral genome can wait for the NE to break down during mitosis.

### Regulated transport of viral RNA genomes using NPCs

For reverse-transcribing viruses and orthomyxoviruses that replicate their genomes in the nucleus but assemble capsids (or nucleocapsids, NC) in the cytoplasm, the NPC represents the preferred route for genome nuclear egress (9, 19–25). NPCs are large, cylindrical protein complexes that, in eukaryotes, consist of more than 50 proteins that collectively span both bilayers of the NE, generating an aqueous portal (4, 26). The NPC exhibits eightfold symmetry, with a polarized configuration wherein eight cytoplasmic fibrils extend into the cytoplasm and a networked substructure known as the “nuclear basket” extends inward into the nucleus from the NPC’s base (27, 28). The NPC consists of proteins known as nucleoporins (“Nups”), with several Nups bearing phenylalanine/glycine-rich repeat regions known as “FG repeats” or “FG fibers” that extend into the central channel of the NPC, creating a hydrophobic sieve with hydrogel-like properties (29–31). Traditionally, the NPC central channel is thought to be ~40 nm in diameter, allowing for free diffusion of small molecules and protein cargoes of under ~40 kDa (32, 33). Compellingly, however, recent high-resolution comparative structural studies using cryo-electron tomography suggest that NPCs can actually be dynamic, capable of both contracting or expanding the pore to diameters as large as ~60 nm (34–37) (Fig. 1A).

Transit across the NPC represents the major route of cellular nucleocytoplasmic trafficking during interphase and is, it follows, the best-studied mode of viral genome nuclear export. Studies of retroviruses, in particular HIV-1, have contributed a wealth of discoveries pertinent to understanding transport using the NPC. In virions, retroviruses carry two copies of an RNA genome intermediate that, during early infection, are reverse-transcribed to generate a single double-stranded DNA genome intermediate known as the provirus that is subsequently integrated into host chromosomal DNA (38). Proviral genome integration is an obligatory stage in the retroviral life cycle, with new RNA genomes subsequently generated through the virus’s hijacking of host RNA Pol II using a viral promoter-enhancer region encoded within the provirus’s 5′ long terminal repeat region. Post-synthesis, ~9 kb full-length retroviral pre-mRNAs are either spliced in the nucleus to generate viral subgenomic RNAs or exported through the NPC, introns-intact (hereafter referred to as “intron-containing” RNA or icRNA), where they can serve as viral mRNAs encoding Gag (and typically Gag-Pol) structural proteins or viral RNA genome substrates that undergo dimerization and are then bound by Gag’s NC domain for genome packaging during virion assembly (9, 24, 39–41). In the context of viral RNA nuclear export, HIV-1 is particularly complex in that the virus generates three major pools of RNA variants using alternative splicing including ~2 kb multiply-spliced mRNA transcripts that encode the viral Tat, Rev, and Nef proteins, ~4 kb partially spliced icRNA variants that encode the Vif, Vpr, and the Envelope proteins, and ~9 full-length icRNAs that, as noted above, serve dual functions in the cytoplasm as either mRNAs encoding Gag and Gag-Pol, or viral genomes (7, 39, 41).

To deliver icRNAs to the cytoplasm, different retroviral subfamilies are remarkable in that they exploit at least two independent host NPC-mediated RNA genome nuclear egress pathways, regulated by either (i) the protein export factor Exportin-1 (XPO1, also known as CRM1) or (ii) Nuclear Export Factor 1 (NXF1, also known as Tap) that is broadly implicated as the major regulator of bulk host mRNA nuclear export (22, 23, 25, 42) (Fig. 1A). Indeed, the XPO1/CRM1 pathway was largely discovered thanks to early studies of the HIV-1 Rev protein, which demonstrated that Rev is essential for viral full-length and partially spliced icRNA nuclear export [binding to a viral RNA structure known as the Rev response Element (RRE)] (25, 43–47), and that Rev’s export activity mapped to a discrete, transferable, hydrophobic (“leucine-rich”) peptide motif (LQLPPLERLTL) dubbed its nuclear export signal (NES) (25, 47–50). By contrast, the multiply-spliced 2 kb HIV-1 mRNA transcripts lack the RRE and are both Rev- and XPO1-independent.

The functional relevance of Rev’s NES was revealed years later with the characterization of XPO1, a host transport receptor that binds Rev’s NES domain and, in the absence of infection, regulates the nuclear export of hundreds of cellular proteins that encode analogous NES peptides (51–53). XPO1’s transport directionality was discovered to be regulated by Ran, a GTPase that, in the nucleus, exists in a GTP-bound state, with Ran-GTP binding activating XPO1 for NES cargo-binding, prior to cargo export through the NPC (54, 55). XPO1 cargoes were subsequently released in the cytoplasm in response to Ran-GTP hydrolysis, an activity restricted to the cytosol that triggers export complex dissociation. As detailed in the next section, multiple retroviral subfamilies encode Rev-equivalent proteins, with most, if not all, thought to encode functional NES domains and operate through a conserved mechanism that involves multimerization on viral icRNA structures known as “export elements,” thereby forming multi-NES viral ribonucleoprotein complex (vRNP) export complexes (cooperative export model, Fig. 1A) and subsequent XPO1- and Ran-driven transport to the cytoplasm. While predominantly a protein exporter, XPO1 has also been demonstrated to export host RNA molecules including U family small nuclear RNAs, a subset of ribosomal RNAs (e.g., 5S), and some host mRNAs that encode AU-rich response elements, invariably regulated by NES-encoding adaptor proteins. While there are no known cellular Rev homologs, it is worth pointing out that a subset of host RNA-binding proteins such as HuR may similarly multimerize in association with RNA elements to form XPO1-dependent multi-NES RNP export complexes (56).

Not all retroviruses encode Rev-like proteins, instead employing RNA structural elements generally known as “constitutive transport elements” (CTEs) that hijack the more conventional NXF1 RNA export pathway through direct recruitment of NXF1 to the viral RNA (i.e., no need for a viral adaptor protein). The best-characterized CTE, to date, is a discrete, transferable, *cis*-acting RNA element found in Mason-Pfizer Monkey Virus (M-PMV), which binds to a heterodimer of NXF1 in association with its cofactor NXT1 (also known as p15), with these coordinated interactions thought to route M-PMV icRNA genomes into the cytoplasm through a pathway similar, if not identical, to that utilized by the bulk of host spliced mRNAs (41, 57, 58) (Fig. 1A). During host mRNA synthesis, stepwise checkpoints intrinsic to 5′ 7 mG pre-mRNA capping, splicing, and 3′ end processing regulated by the transcription-export machinery govern recruitment of NXF1, with NXF1 binding mRNA in the nucleus in response to recruitment transcript-bound adaptor proteins that function in concert with splicing factors (4, 10, 59–62). mRNA-NXF1 complexes are subsequently routed through the NPC, mediated by interactions between NXF1’s central nuclear transport receptor domain and central channel FG-Nups, analogous to XPO1. Once in the cytoplasm, similar to XPO1, NXF1 undergoes structural changes that allow it to disengage and release its mRNA cargo [reviewed in references (4, 63)]. The M-PMV CTE differs from conventional cellular mRNA in that the CTE binds to NXF1 directly, likely in the absence of adaptor proteins, and how NXF1 is subsequently released from viral RNA (if it is) remains unknown (18, 64, 65). CTE-like elements have also been described for additional retroviruses that lack Rev-like accessory proteins including murine gammaretroviruses (66–68) and avian alpharetroviruses (69, 70).

Compellingly, orthomyxoviruses such as influenza A virus are also adapted to exploit the XPO1 pathway for genome nuclear export. Unlike retroviruses, orthomyxoviruses are negative-strand RNA viruses that encapsidate multiple RNA genome segments known as vRNP complexes that are synthesized in the nucleus independently of the host transcriptional machinery (19, 20, 71). Individual vRNP complexes are subsequently exported to the cytoplasm, mediated through stepwise interactions between the viral nucleocapsid (N) protein, viral M1 (matrix) protein, and viral nuclear export protein that encodes two independent NES domains thought to mediate recruitment of XPO1 (21, 72, 73). Interestingly, late in infection, influenza may also drive significant enlargement of the nuclear pore through the protease activity of apoptotic caspases, allowing for free outward diffusion of vRNPs, independently of XPO1 (74).

### NPC-independent routes of viral genome nuclear egress

A second fascinating mode of viral genome nuclear egress is avoidance of the NPC altogether to transit the NE, best described for the alphaherpesviruses herpes simplex virus type 1 (HSV-1). HSV-1 encapsidates large (>100 kb) DNA genomes in the nucleus prior to nuclear egress, forming capsids that are too large to traverse an NPC (Fig. 1B). The inner face of the NE, known as the lamina, is coated with a dense network of proteins called lamins (Lamin A/C and Lamin B), which provide structure to the nucleus during interphase (75, 76) but are displaced during mitosis prior to NE breakdown in response to phosphorylation, mediated by isoforms of cellular protein kinase C (PKC) (77, 78). In order to access the NE for egress, HSV-1 capsids both activate host PKC and employ their own encoded serine/threonine kinases (e.g., HSV-1 Us3) that phosphorylate lamin proteins, allowing for viral capsid proteins (e.g., the HSV-1 UL31 and UL34) to associate with the inner face of the NE and drive budding (13, 14, 79–81). It is worth noting that this pathway resembles a budding-driven mechanism for NPC-independent nuclear export of large cellular RNPs characterized in *Drosophila* neuronal cells, suggesting evolutionary parallels (82, 83). However, despite sometimes exhibiting large sizes (e.g., retroviral genomes), to date, NE budding has not been observed for viral RNA cargoes. By contrast, baculoviruses, large DNA viruses found in insects that also packaged their genomes in the nucleus, may exploit a similar pathway (84, 85).

Additionally, there is potential for viruses to circumvent the NE barrier entirely through either driving NE dissolution in conjunction with virus-induced cytopathic effects or, for viruses infecting cells that are actively dividing, leaving the nucleus during NE breakdown during mitosis (Fig. 1C). Some non-enveloped DNA viruses including adenoviruses and parvoviruses are thought to trigger NE dissolution during late-stage infection, thereby allowing for direct escape of their genomes, already encapsidated in the nucleus, to leave the cell in conjunction with apoptosis or cell lysis (15, 86). Whether viruses exploit cell division for genome nuclear egress is less clear; however, we recently discovered using live cell imaging that retroviral CTE-bearing icRNA transcripts can associate with centrosomes that form mitotic spindle poles during mitosis in cancer cells, subsequently “riding” each pole to the cytoplasm of each daughter cell (18). Moreover, while hepadnaviruses such as hepatitis B virus (HBV) are traditionally thought to export their RNA genomes (and perhaps intact capsids) out of the nucleus through the NPC (87–90), we also recently demonstrated that HBV capsids that assemble in the nucleus can be translocated to the cytoplasm in conjunction with hepatocyte cell division, subsequently retained in the cytoplasm through a yet-to-be-defined tethering mechanism (17).

### Links between viral genome nuclear export pathways and cytoplasmic fate

For retroviruses, the seemingly simplest route for genome nuclear export from the nucleus to the cytoplasm would be to directly couple viral RNA genomes to nuclear export receptors, as illustrated by M-PMV with NXF1 bound to the CTE. Accordingly, why a subset of retroviruses including *lentiviruses* such as HIV-1, *deltaretroviruses* such as the human T lymphotropic virus (HTLV), and some *betaretroviruses* (e.g., mouse mammary tumor virus) evolved to encode Rev-like proteins that serve as adaptors to access the XPO1 pathway is not immediately clear. Indeed, many studies have confirmed that the RRE in HIV-1 can be replaced functionally to restore icRNA nuclear export by one or more copies of a CTE-like export module (91–94).

The viral transcript coding for HIV-1 Rev, like most Rev-like proteins, is completely spliced and thus exported to the cytoplasm through the NPC similar to most cellular mRNAs that use the NXF1/NXT1 pathway. Thus, in its simplest interpretation, Rev-like proteins provide retroviruses with a means to switch from early Rev-independent, NXF1-driven (i.e.*,* spliced) gene expression to “late” icRNA-linked gene expression that yields structural proteins (i.e., Gag, Gag-Pol, and Envelope). Following this view, HIV-1 RNA splicing will proceed in the nucleus until Rev reaches a critical concentration, then switching the program to icRNA export and, potentially, acting to suppress splicing machinery (25, 95). In infected cells, such a delay may provide a crucial time window for the virus to reprogram the cell in important ways prior to virion production, e.g., a key role of Nef (an early, Rev-independent viral gene product) is to downregulate host proteins that can reduce infectious virus production including CD4, BST2/Tetherin, and SERINC3/5 (96–100). There is also evidence that XPO1 provides HIV-1 with a more reliable route of nuclear export and icRNA cytoplasmic utilization in the face of cellular activation or induction of virus-induced cell stresses that otherwise impact the integrity of the NPC or abrogate host mRNA translation, ensuring that viral genome export and structural protein synthesis continue to provide for ongoing robust virion production (12, 101–105).

It is also important to consider that, once in the cytoplasm, the ultimate purpose of genome nuclear export is to provide the virus with a means to deliver genomes to the virion egress pathway. Accordingly, successful transit across the NPC for retroviruses and orthomyxoviruses would need to position genomes in the subcellular domains that are most appropriate for ensuring efficient encapsidation and/or access to the cellular egress machinery. Based on these considerations, we have previously employed comparative live cell imaging to demonstrate that HIV-1 genomes leaving the nucleus through the Rev/XPO1-driven pathway tend to flood the cytoplasm to readily accumulate in the cell periphery, where virus particles assemble (18, 106, 107). In contrast, CTE-driven M-PMV genomes or HIV-1 model transcripts where the RRE was replaced with multiple copies of the CTE were more frequently associated with microtubules, accumulating transiently at the microtubule-organizing center (MTOC) (18). Based on these results, we have speculated that for retroviruses, HIV-1’s Rev-driven genome export and cytosolic diffusion is a feature consistent with it representing a “C-type” retrovirus that assembles virus particles at the plasma membrane, in contrast to “D-type” retroviruses such as M-PMV that assemble capsids preferentially in the cytoplasm prior to microtubule-dependent transport to bud sites at the plasma membrane (18,108). That said, prior studies by Lehmann et al. (109) and Lévesque et al. (110) linked HIV-1 RNA trafficking to intracellular membranes as well as the cytoskeleton, while RRE- or CTE-dependent links to microtubules and the MTOC were less apparent in a recent imaging-based study led by Chen and colleagues (111). Accordingly, it is not unlikely that links between nuclear export and cytoplasmic transport pathways are context-dependent and merit further study.

Also consistent with nuclear export pathways licensing retroviral genomes for cytoplasmic fate, multiple additional studies have indicated roles for the HIV-1 Rev in governing the efficiency of icRNA translation, genome packaging, and even induction of cell signaling linked to inflammatory cytokine production (112–116). However, whether these Rev-linked phenotypes are due purely to trafficking/localization as opposed to intrinsic structural features (e.g., epitranscriptomic modifications) remains unresolved. Moreover, it is worth noting that, while less common, some retroviruses are thought to hijack XPO1 for icRNA nuclear export using Rev alternatives such as host-derived RNA adaptor proteins (e.g., HuR for prototype foamy virus; a spumaretrovirus) (117) or to use hybrid, Rev-independent approaches wherein both NXF1- and XPO1-dependent pathways are utilized to demarcate viral icRNA pools for dedicated purposes (i.e., translation vs packaging) in the cytoplasm (e.g., as proposed for murine leukemia virus and Rous sarcoma virus) (118, 119).

## IT TAKES (AT LEAST) TWO TO TANGO: REV-XPO1 COOPERATIVE INTERACTIONS DRIVE THE FORMATION OF FUNCTIONAL HIV-1 ICRNA EXPORT COMPLEXES

**Fig 2:**
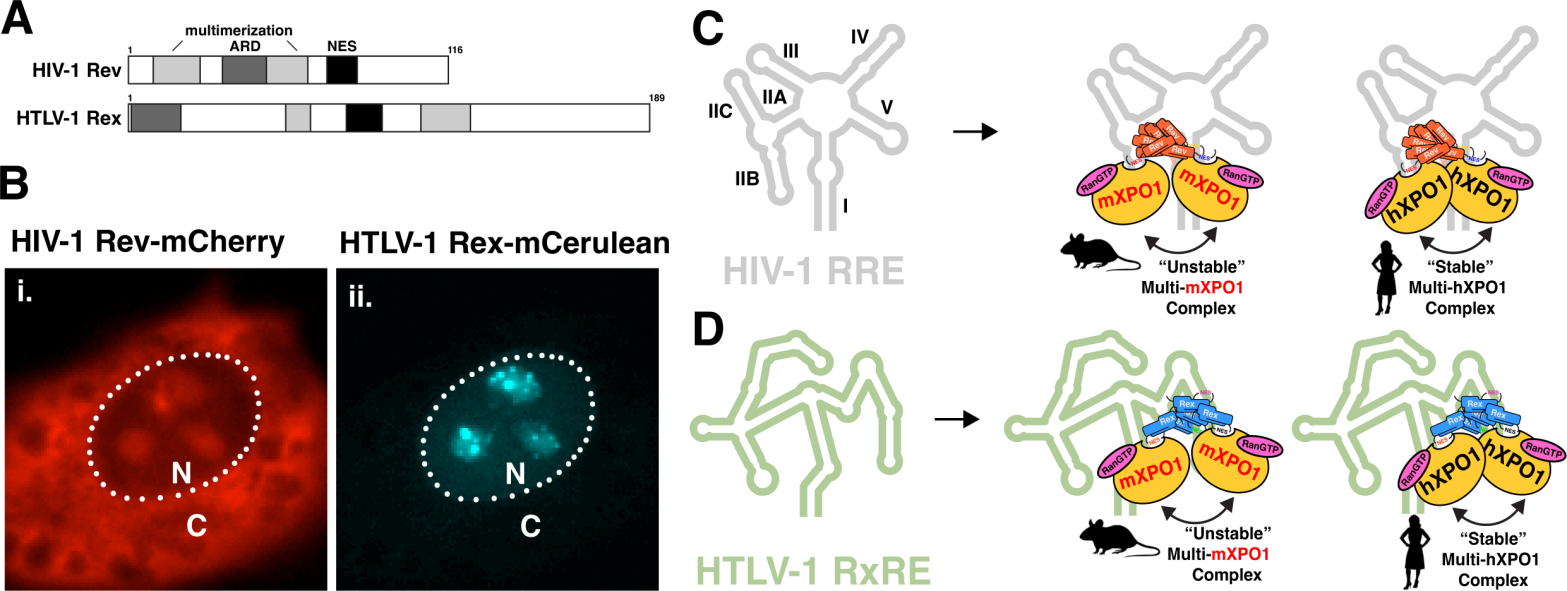
Rev-like proteins orchestrate the cooperative assembly of retroviral nuclear export complexes. (**A**) Distinct organization of key functional domains for HIV-1 Rev (top) and HTLV-1 Rex (bottom). RNA binding and nuclear localization signals are encoded within arginine-rich domains (ARD, dark gray). Oligomerization domains (light gray) confer homooligomeric interactions. Nuclear export signals (black) enable active transport from the nucleus to the cytoplasm using the XPO1 pathway. (**B**) Distinct subcellular localization of co-expressed and fluorescently tagged HIV-1 Rev (i) and HTLV-1 Rex (ii) fusion proteins. To study subcellular localization, HeLa cells were transfected to co-express HIV-1 Rev-mCherry (red) and HTLV-1 Rex-mCerulean. HIV-1 Rev-mCherry was diffusely enriched in the cytoplasm and in the nucleolus. By contrast, co-expression was predominantly nuclear and enriched at specific nucleolar regions. N, nucleus; C, cytoplasm; dotted line indicates the nuclear envelope boundary. (**C**) Distantly related retroviral nuclear export complexes are likely to rely on a human-encoded XPO1 dimer interface. HIV-1 Rev monomers bind to and multimerize on the HIV-1 RRE (RRE stem loops are indicated). Rev/RRE complexes recruit the host nuclear export machinery, with XPO1 bound by RanGTP. Efficient vRNP assembly and export are dependent on the human-specific XPO1 patch element that is proposed to mediate an XPO1-XPO1 dimer interaction. In mouse cells, mXPO1 dimerization and Rev-driven vRNA export are inefficient. (**D**) Model depicting HTLV-1 Rex/RxRE nuclear export complexes. Like HIV-1 Rev, the activity of HTLV-1 Rex is defective in mouse cells, suggesting that the hXPO1 patch element is also important for Rex-mediated viral gene expression. Thus, HTLV-1 nuclear export complexes are predicted to form structures on the RxRE scaffold that expose multiple NES domains to achieve an XPO1 dimer configuration akin to those determined for HIV-1.

Retroviral Rev-like proteins typically feature a conserved set of required functional domains, including a nuclear localization signal (NLS), NES, as well as an RNA binding domain (RBD) and oligomerization domains (ODs) (Fig. 2A, HIV-1 Rev, top; HTLV-1 Rex, bottom). While the nucleocytoplasmic shuttling and RNA binding activities are fundamentally necessary for a shuttling RNA adaptor protein, the additional requirement of ODs for Rev-like proteins to license icRNA nuclear export emphasizes, in part, that cooperativity is a principal feature required for the formation of export-competent vRNP assemblies (120). When Rev levels reach a sufficient threshold, Rev in the nucleus binds to and oligomerizes on nascent, RRE-containing viral RNAs (46, 121, 122). While it is appreciated that higher-order Rev oligomerization requires the RRE and is essential to transport these substrates, Rev proteins alone can multimerize in cells in the absence of the RRE and even form long filamentous assemblies *in vitro* (123–126). To date, two Rev monomers can associate using at least three distinct interfaces (often designated as “A-A,” “B-B,” or “C-C” configurations) (127–129). Each Rev dimer forms a contact point at discrete positions along the resolved helix-loop-helix N-terminus, about which the two monomers may adopt different cross-angle orientations. Models indicate each interface may participate at different points in the Rev/RRE assembly process and that some dimer configurations form independently of the RRE substrate (130).

The prototypical HIV-1 RRE is a 351-nucleotide (nt) RNA element defined by a long base stem (SL-I), topped by a branched structure of stem-loops that can adopt different overall architectures (SL-II to SL-IV) (130–137). The branch point of stem-loop IIB (SL-IIB) is the conserved initial, high-affinity HIV-1 Rev binding site (44, 45, 138–140). The SL-IIB site can accommodate Rev monomers or dimers, although it is proposed that SL-IIB bound by a Rev dimer stabilizes a critical initial contortion of the Rev/RRE complex that facilitates the successive, cooperative incorporation of Rev molecules into the vRNP. Rev oligomerization extends from SL-IIB to SL-I, bridging the two stems and resulting in a vRNP complex with increased net avidity (127, 130, 133, 141). Structural changes to the RRE and bound Rev oligomer are thought to then culminate in NES-dependent recruitment of XPO1 bound by its Ran-GTP co-factor (50, 51, 142–145).

At maturity, HIV-1 export-competent vRNPs are estimated to contain between 6 and 13 Rev monomers, although the minimal number of Rev monomers is unclear. Rev’s C-terminus, which contains the NES, is intrinsically disordered and was not determined in initial Rev structures. However, filamentous Rev assemblies captured in more recent crystal structures, along with interaction maps based on evolutionary coupling analyses, indicate Rev’s C-terminus may become ordered or harbor additional protein interaction domains (129, 146, 147). Removed from its native context, the structure of Rev’s NES domain has been resolved in co-crystals of XPO1 nuclear export complexes (148–150). The disordered nature of Rev’s C-terminus may suggest that flexibility contributes to Rev’s recruitment of XPO1.

As noted above, due to XPO1’s affinity for components of the NPC, XPO1-bound Rev/RRE complexes are thought to exit the nucleus through the NPC and disassemble in the cytoplasm (151). Dissociation of the Rev/RRE is triggered by hydrolysis of the Ran-bound GTP to GDP, which destabilizes the XPO1 binding pocket that accommodates NES domains (152, 153). What remains of the Rev/RRE vRNP following dissociation from XPO1 is unclear, although Rev and divergent Rev-like proteins have been suggested to remain associated with icRNAs following nuclear export and, as noted above, may influence cytoplasmic events (113, 154–159).

Taken together, Rev’s timing of its interactions with other Rev molecules, viral RNA, and XPO1 must be tightly regulated in order for Rev to do its job. In the following section, we summarize the challenges that Rev faces in subcellular transport and how Rev-Rev and Rev-XPO1 interactions are thought to be coordinated in space and time. We focus predominantly on HIV-1 Rev, which is best studied, but also discuss the remarkable conservation of this pathway among all other known *lentiviruses* and *deltaretroviruses*.

### Rev subcellular trafficking: balancing antagonistic forces of a strong NLS and strong NES

Upon its synthesis, Rev is licensed for nuclear import by its arginine-rich NLS that overlaps its RNA-binding domain. Rev utilizes Importin-β (KPNB1), or other karyopherin-family nuclear transport receptor pathways (e.g*.,* Transportin or Importin-5 or -7 receptors), for active translocation into the nucleus (160). Interestingly, HIV-1 Rev is of the class of NLS-containing cargos that does not require the KPNA1 (Importin-α) adaptor for binding to KPNB1 (161, 162). KPNB1 engages cargo proteins in the cytoplasm and engages with NPC proteins to enter the pore. In the nucleus, Ran-GTP binding to KPNB1 triggers cargo release (163).

Once delivered to the nucleus, Rev is frequently observed enriched in the nucleolus, a stratified, phase-separated nuclear condensate that serves as the hub for ribosome biogenesis (164, 165). The localization of HIV-1 Rev to the nucleolus is dependent on active transcription, nucleolar resident proteins (e.g., B23, also known as nucleophosmin), and discrete nucleolar targeting sequences within the NLS also found in divergent Rev-like proteins (166–172). Some studies have indicated that nucleolar trafficking is critical for Rev function, wherein the nucleolus may serve as a “staging area” for Rev to accumulate to levels sufficient for capturing and exporting icRNAs, and perhaps recruiting nucleolar-associated co-factors that participate in Rev activity (167, 168, 173, 174). Consistent with this notion, other Rev-like proteins such as HTLV-1 Rex exhibit a preference for localization to the nucleolus; however, Rex exhibits a subnucleolar localization pattern distinct from Rev that suggests differential protein or RNA interactions (Fig. 2B). On the other hand, our prior single-cell imaging studies have indicated a lack of correlation between HIV-1 Rev steady-state nucleolar localization and icRNA export, so that association with the nucleolus may be transient or, in the least, is not entirely predictive of activity (175).

In an effort to understand the regulation of Rev’s subcellular localization, we recently employed an optogenetic tool (light-activated NES exposure) (176) to demonstrate that Rev’s nucleolar retention is dependent on the ability of Rev to suppress interactions between XPO1 and its encoded NES (a concept known as “NES masking”) (107). RRE-independent Rev-Rev interactions were key determinants of this activity, suggesting that the formation of low-order Rev multimers in the cytoplasm acts to cloak Rev’s NES domains, thereby suppressing XPO1 interactions to promote the activities of Rev’s overlapping NLS/RBD domain. Based on these data, we have proposed that the directionality of Rev’s subcellular transport is governed by differential interactions among Rev monomers, with dimerization and NES masking promoting KPNB1 association for nuclear delivery, followed by higher-order multimerization on the RRE to expose the NES and trigger XPO1 association (107). Consistent with a model wherein Rev is trafficked to the nucleus as a preformed, NES-masked dimer, it was recently confirmed that a single KPNB1 transport complex is able to accommodate two Rev proteins (177).

### Cooperative Rev-Rev interactions for multi-XPO1 complexes: lessons from the mouse

Structure-function studies in some cellular settings have also demonstrated that interactions between XPO1, Rev, and cognate viral RNA export elements can be insufficient for icRNA gene expression (175). For example, soon after the discovery of HIV-1 Rev and its primary role in HIV-1 gene expression, it was shown that Rev and HTLV-1 Rex functions were severely attenuated in cells of rodent origin (e.g., from mice, rats, and hamsters) (178). By fusing rodent and human cells, icRNA-dependent gene expression was restored, implying that rodent cells lack a key factor needed for Rev and Rex’s icRNA nuclear export functions (178–180). Following several advances in our understanding of the Rev protein domains, karyopherin-dependent nucleocytoplasmic trafficking, and the mapping of a Rev co-factor to human chromosome 2, it was demonstrated that icRNA-dependent gene expression could be restored by supplementing rodent cells with the human ortholog of XPO1 (hXPO1), but not its rodent counterparts, thereby pinpointing XPO1 as a species-specific determinant of Rev function (181–184). Mammalian XPO1 orthologs are conserved, with the human and mouse versions encoding ~98% protein identity. Mapping studies revealed three XPO1 residues (T411, V412, and S414 in mice; P411, M412, and F414 in humans; constituents of HEAT repeat 9A) that define a surface-exposed, species-specific “patch-like” surface element hypothesized to represent a binding interface (183, 184). Importantly, these residues are distinct from the known and species-conserved binding cleft where NES peptides are bound by XPO1.

How the XPO1 species-specific domain (hereafter referred to as “patch element”) contributed to the success of Rev/RRE nuclear export was not clear. Based on Rev being primarily localized to the cytoplasm in certain nonpermissive rodent cell lines, we hypothesized that interactions between Rev and mouse XPO1 (mXPO1) were overactive, leading us to append additional NLS or NES domains to functional fluorescently tagged derivatives of Rev to bias its trafficking and residence time in the nucleus (175). An appended second NLS shifted Rev localization to the nucleus of mouse cells but did not significantly affect its activity. Surprisingly, however, a second appended NES domain completely restored Rev activity in mouse cells even in the absence of hXPO1, suggesting that insufficient interactions between Rev/RRE complexes and mXPO1 underpinned the rodent-specific defect. We hypothesized that multiple Rev NES domains must be needed to recruit multiple mXPO1 molecules to the Rev-bound icRNAs and generated data in support of this hypothesis by adding a second RRE to the icRNA, observing a similar rescue of Rev activity in rodent cells. Taken together, we proposed that Rev’s multimerization on the RRE represents a step-wise, cooperative mechanism regulated by the formation of a multi-NES complex, with Rev’s multi-NES complex acting to recruit more than a single XPO1 protein in order to produce a functional RNA export complex (Fig. 2C).

Additional insights have been gleaned from structure-function studies using modified HIV-1 Rev proteins that illustrate additional features of their cooperative action. For example, Hoffmann and colleagues used forced dimerization of distinct Rev mutants to demonstrate that not all Rev monomers in the multi-Rev complex need to encode a functional NES domain in order to mediate efficient export (185). Another study by Furnes et al. showed that the addition of an RBD/NLS domain from HTLV-1 Rex appended to Rev’s N-terminus restored activity to an HIV-1 Rev mutant with inactivating substitutions in one of the two ODs (186). We have also shown that inactivation of the native Rev NES domain in the context of a second, appended NES at the C-terminus of a Rev-mCherry fusion protein (i.e., RevM10-NES) maintained nuclear export activity in human cells but was not responsive to hXPO1 in mouse cells (107), indicating that NES positional context is also a determinant of functional Rev-XPO1 interactions (187, 188). Combined, these studies demonstrate that a function of Rev’s cooperative association with the RRE is to impart flexibility as to the ways that Rev protein subunits can activate the nuclear export of a vRNP. Importantly, however, the relative contributions of each functional domain to vRNP integrity remain unclear.

In this context, Booth and colleagues published a key study that also investigated the species-specific XPO1 interaction with Rev/RRE complexes, but instead using a biochemical and structural approach *in vitro* (145). The results of this study were consistent with the species-specific XPO1 patch element representing a self-binding interface between two adjacent XPO1 molecules, formed upon engagement with the multi-NES Rev-RRE complex. Gel mobility shift and size-exclusion chromatography experiments demonstrated that Rev/RRE assemblies formed high molecular weight complexes, consistent with the recruitment of two XPO1 proteins, more readily with the human version of XPO1 compared to its mouse ortholog (Fig. 2C). 3D reconstruction of single particle electron microscopy images captured of the XPO1-bound RRE complexes revealed that the species-specific XPO1 patch elements were positioned facing each other, with the density of the Rev/RRE complex situated near this junction. Furthermore, each XPO1 NES binding cleft was oriented to either side of this junction, implying that two discrete Rev monomers provide independent anchoring points mediated through NES-XPO1 binding (Fig. 1A and 2C). This study indicated that the cooperative interactions required for the Rev/RRE assembly synergize with cooperative interactions between XPO1 monomers. Additional structure-function studies have demonstrated that differences in the Rev interaction between hXPO1 and mXPO1 can be detected even in the absence of the RRE (189). These observations raise the possibilities that Rev binding to cellular RNA scaffolds (123, 190) are sufficient to drive XPO1 dimerization and that auxiliary Rev interactions with XPO1 (143) exist that are similarly sensitive to the species-variable regions of XPO1.

As noted above, several early studies demonstrated that the nuclear export activity of HTLV-1 Rex is similarly restricted in rodent cells, suggesting that Rex may coordinate the formation of a vRNP that depends on the same species-specific, cooperative XPO1 interaction observed for the HIV-1 Rev/RRE complex (Fig. 2D, depiction of theoretical model) (191–193). Interestingly, more recent reports extend this finding to lentiviruses of non-human origin (e.g., feline immunodeficiency virus) (184, 189). While structural information relevant to the nuclear export complexes outside of the HIV-1 system is limited, the apparent dependency on species-specific XPO1 features suggests that Rev/Rex cooperativity and higher-order XPO1 interactions are generalizable features of export-competent retroviral vRNPs.

### Evolutionary conservation of multi-NES genome nuclear export machines

The discovery of HIV-1 Rev has been complemented by studies of analogous Rev-like systems found in evolutionary distant retroviruses. Beyond the aforementioned similarities with the HIV-1 Rev at the functional level, HTLV-1 Rex was observed to be capable of transactivating icRNA-dependent nuclear export using the HIV-1 RRE despite a lack of sequence identity (194, 195). Considering the distinct organization of the core functional domains (i.e.*,* NLS and NES) in Rex (196) (Fig. 2A), we interpret this interoperability to be a testament to the plasticity and shared evolutionary origin of XPO1-dependent retroviruses. However, there are insightful limitations to the plasticity of Rev/RRE complexes, e.g., although HTLV-1 Rex can function with the HIV-1 RRE, an inverse interaction (HIV-1 Rev added to the HTLV-1 RxRE) is non-functional (194). Moreover, mapping studies illustrated that HIV-1 Rev and HTLV-1 Rex exhibit distinct, high-affinity binding sites on the HIV-1 RRE that can be functionally separated (i.e*.,* deleting SL-IIB led to inactivation of HIV-1 Rev activity while leaving HTLV-1 Rex activity intact, and vice versa) (197, 198).

While most examples of interoperability have been determined for extant, complex retroviruses (195, 199, 200), more extreme examples have also been identified. Up to 8% of the human genome comprises sequences of retroviral origin (201). A small subset of these sequences resembles partially intact complex retroviruses, such as the human endogenous retrovirus type K (HERV-K, also known as human mouse mammary tumor virus like-2, or HML-2), which encode identifiable Rev-like proteins (e.g., HERV-K Rec also known as K-Rev) and response elements (e.g., the HERV-K RcRE also known as the K-RRE) (202–205). HERV-K relatives have been integrating into the human germline for the last ~100 million years, and while most endogenous human retroviruses are inactive due to gene decay or recombination, some more recent integrants retain residual nuclear export activity (201, 206). RcRE-containing HERV-K transcripts are expressed and remarkably, despite significant evolutionary separation, can be exported from the nucleus by HIV-1 Rev (207). Recent structural studies show that the RcRE retains stem-loop branching topologies that resemble the key Rev binding site on the HIV-1 RRE (208). Like the relationship with HTLV-1 Rex, Rec cannot, in turn, transactivate HIV-1 RRE-dependent gene expression, reemphasizing the selective nature of the HIV-1 RRE. Beyond retroviruses, Diaz and colleagues reported that the HSV-1 protein US11, which has a known role in regulating HSV-1 gene expression, could bind to and confer a partial rescue to icRNA-dependent gene expression via the HTLV-1 RxRE (209, 210). Rev-like analogs outside of retroviruses are rare but may provide additional clues to the ways in which vRNPs destined for the cytoplasm are formed and exit the nucleus.

Taken together, functional studies using minimally defined RNA substrates demonstrate that highly divergent Rev-like proteins and a given viral RNA element can assemble into export-competent vRNPs. However, aside from the HIV-1 Rev/RRE example, we have only a cursory understanding of the assembly steps needed to form alternative export-competent vRNPs. Future studies that provide structural insight should shed light on how select assemblies culminate in successful RNA export, while others do not. Indeed, Rev-like proteins are diverse in primary sequence and molecular mass and importantly have divergent configurations of their functional domains (211). While some organizational differences likely reflect varying constraints due to overlapping open reading frames or other RNA features (212), we envision that differences could also be tailored to new functions specific to the needs of individual viruses to regulate specific nuclear (i.e., transcription and splicing) or cytoplasmic events (i.e.*,* translation and genome encapsidation).
